# Brucella seroprevalence in cattle near a wildlife reserve in Kenya

**DOI:** 10.1186/s13104-017-2941-x

**Published:** 2017-11-25

**Authors:** Sofie Enström, Daniel Nthiwa, Bernard Bett, Amanda Karlsson, Silvia Alonso, Johanna F. Lindahl

**Affiliations:** 1 Department of Clinical Sciences, Swedish University of Agricultural Sciences, Uppsala, Sweden; 2 Department of Biosciences, International Livestock Research Institute, Nairobi, Kenya; 3 Department of Biological Sciences, University of Embu, Embu, Kenya; 4 Department of Biosciences, International Livestock Research Institute, Addis Ababa, Ethiopia; 5 Department of Medical Biochemistry and Microbiology, Uppsala University, Uppsala, Sweden

**Keywords:** Zoonosis, *Brucella abortus*, Livestock-wildlife interface, Pastoralism, Africa

## Abstract

**Objectives:**

Brucellosis is caused by bacteria from the genus *Brucella* which infect human and domestic animals as well as wildlife. The Maasai Mara National Reserve has vast populations of wild ruminants such as buffaloes and wildebeest which could contribute to the risk of brucellosis in livestock, and the surrounding pastoralist communities grazing cattle in and around the reserve may be exposed to a higher risk of zoonotic diseases like brucellosis due to the close contact with livestock. In this study, cattle from three villages at varying distance from the reserve, were screened for antibodies against *Brucella abortus*.

**Results:**

In total, 12.44% of 225 sampled animals were seropositive, with more females (15%) infected than males (5%). Seroprevalence was higher in livestock closer to Maasai Mara with the cattle in the village Mara Rianta having an odds ratio of 7.03 compared to Endoinyo Narasha further away (95% CI 1.4–11.1, p = 0.003), suggesting that a closer contact with wildlife may increase the circulation of infectious diseases between livestock and wildlife. Symptoms consistent with brucellosis were reported to occur in both humans and animals, and we thus conclude that brucellosis may be an important problem, both for the health and the economy.

**Electronic supplementary material:**

The online version of this article (10.1186/s13104-017-2941-x) contains supplementary material, which is available to authorized users.

## Introduction

In sub-Saharan Africa, livestock are essential for the livelihood of many families, and the health of livestock, humans and their economic welfare are closely linked [1]. Zoonotic diseases create a double burden on the households with both human and animal morbidity [2]. Brucellosis is a severe disease for farmers since it does not only cause serious chronic disease in humans and suffering in their animals, but also a decreased production since the disease is associated with abortions and reproductive failures in the livestock [3].

In cattle the infection is predominantly caused by *B. abortus,* sometimes by *B. melitensis* (more common in small ruminants) and occasionally by *B. suis* [4–6]. Different wildlife species, especially ruminants, provide a potential reservoir of brucellosis, which has been suggested to increase the risk infections in livestock [7].

In Kenya, awareness of brucellosis is low among livestock-keepers and healthcare staff, and because of the general symptoms and the limited availability of laboratory facilities, diagnosis is not easy [8, 9]. Moreover, some of the areas with high cultural and economic dependence on livestock are close to wildlife protected areas, such as the Maasai Mara national reserve. The aim of this study was to determine the current seroprevalence of bovine brucellosis and the associated exposure factors for transmission, in three villages close to Maasai Mara.

## Main text

### Methods

Three villages (Lemek, Mara Rianta and Endoinyo Narasha) were selected purposively to represent areas with expected differences in livestock-wildlife interaction based on the distance to the national reserve. Both Lemek and Endoinyo Narasha represented areas with less livestock-wildlife overlap. In Lemek there was free grazing; in Endoinyo Narasha sedentary grazing systems in fenced lands were being adopted, while Mara Rianta had high wildlife-livestock interactions due to livestock grazing in the park and communal grazing lands.

The sample size was determined by logistic possibilities. Twenty-five households, all keeping livestock, were randomly selected in each village. Livestock owners were interviewed using a questionnaire translated to Swahili and Maa, where they were asked to answer questions about animal keeping, grazing strategies, herd sizes, experienced signs of illness and other significant details that could be of importance to the study (Additional file 1). The questionnaire was developed by experts at ILRI and pre-tested in the field. Three cattle over 1 year were randomly selected (excluding large bulls and aggressive animals for safety reasons) in each herd for blood sampling. The serum was analyzed in duplicates using an indirect Enzyme-Linked Immunosorbent assay (ELISA); PrioCHECK^®^ *Brucella* Antibody 2.0 ELISA (Thermo Fisher Scientific) according to manufacturer’s instructions. Univariable analyses for risk factors for seropositivity was done using Stata (STATA 14, StataCorp LP, USA). A risk score was calculated adding all risk practices; whether cattle are let to graze inside the national reserve; whether cattle mix with other herds while grazing; whether cattle mix with other herds at watering points; whether cattle share trek route with other herds; whether herds share water point on trek; whether farmer has bought livestock from other farms; whether working personnel visit other livestock holdings; and whether vehicles have access to farm. Correction for multiple comparisons was not done. Data is provided as Additional file 2.

The study had ethical approval from International Livestock Research Institute (ILRI) animal care and use committee (IACUC Approval 2016–20).

## Results

A total of 225 animals were sampled, and 28 of them [12.44%; 95% confidence interval (CI) 7.71–15.41] were seropositive (Table 1). None of the farmers indicated that they had ever vaccinated livestock against brucellosis. More animals, 22.7% (95% CI 14.7–33.3), were positive in Mara Rianta and the odds ratio (OR) was 7.03 (p = 0.003) higher than Endoinyo Narasha, where seroprevalence was 4.0% (95% CI 1.4–11.1), and OR was 2.45 (p = 0.053) higher for Mara Rianta than in Lemek where the prevalence was 10.7 (95% CI 5.5–19.7). Females had a higher risk (OR 4.0, p = 0.046) of being seropositive compared to males, with 25/166 females (15.1%, 95% CI 10.4–21.3) being positive compared to 3/59 males (5.1%, 95% CI 1.7–13.9). All three positive males were sampled in Mara Rianta.

**Table 1:** Distribution of *Brucella* seropositive cattle in Maasai Mara national reserve in Kenya, divided into study sites and sex

	Total	Positive	% Positive	95% CI
Total	225	28	12.44	7.71–15.41
Female	166	25	15.06	10.41–21.29
Male	59	3	5.08	1.74–13.92
Lemek	75	8	10.67	5.50–19.66
Mara Rianta	75	17	22.67	14.66–33.34
Endoinyo Narasha	75	3	4.00	1.37–11.11

Twenty farms (26.7%) had one or more *Brucella*-infected cattle. During the last year, many farmers had experienced abortion (or loss of pregnancy, or stillbirth) in their cows (68, 48 and 36% in Mara Rianta, Lemek and Endoinyo Narasha respectively). In 13 of the infected farms (65%), the farmer reported abortions during the last 1 year. The odds ratio of having had abortion if *Brucella* was present in a farm was 2.2 higher than if *Brucella* was not present (CI 0.7–6.4), and in 34.2% of the farms that have experienced abortion in cattle the last 1 year, the farm was positive for *Brucella*, in even, antibodies against *Brucella* was present in serum from one or more of the three sampled animals (Table 2).

**Table 2:** *Brucella abortus* positive cattle farms in Maasai Mara national reserve (Kenya) and occurrence of abortions

	Farm with abortion	Farm without abortion	Total
Positive farm	13	7	20
Negative farm	25	30	55
Total	38	37	75

All farms had at least one bull for breeding and practiced natural mating. All the participants reported that animals had regular contact with wild ungulates and other cattle. Farms in Mara Rianta were generally larger compared to Lemek and Endoinyo Narasha (Table 3). Clinical pictures compatible with brucellosis were also reported in humans handling livestock. The most common was headache (48, 60 and 64% in Mara Rianta, Lemek and Endoinyo Narasha respectively), muscle, joint and back pain (52, 56 and 64% in Mara Rianta, Lemek and Endoinyo Narasha respectively) and fever (48, 44 and 40% in Mara Rianta, Lemek and Endoinyo Narasha respectively) (Table 3).

**Table 3:** Study on *Brucella abortus* seropositivity in cattle of three villages around Maasai Mara national reserve (Kenya): herd characteristics and results from a questionnaire on risk practices and clinical signs compatible with brucellosis in cattle and in humans handling livestock

	Overall (N = 75)	Lemek (N = 25)	Mara Rianta (N = 25)	Endoinyo Narasha (N = 25)
Cattle herd size	109 (5–400)	97 (15–300)	144 (14–400)	85 (5–300)
Years of operation	34.6 (5–70)	35.32 (15–60)	37.08 (15–70)	31.44 (5–70)
Grazing in reserve	60%	32%	100%	48%
Bought livestock during the last year	79%	88%	64%	84%
Contact with wildlife reported by the farmer				
Ungulates	100%	100%	100%	100%
Predators	76%	52%	100%	76%
Monkeys	35%	0%	36%	68%
Contact with other livestock reported by the farmer				
Cattle	100%	100%	100%	100%
Goats	80%	92%	48%	100%
Sheep	80%	92%	48%	100%
Pigs	0%	0%	0%	0%
Poultry	0%	0%	0%	0%
Signs of illness noticed in cattle				
Fever	4%	8%	4%	0%
Fatigue	0%	0%	0%	0%
Reduced fertility/abortion and/or stillbirth	51%	48%	68%	36%
Decrease in milk production	9%	12%	4%	12%
Mastitis/udder swelling and/or pain	23%	32%	24%	12%
Self-reported signs of illness experienced by people handling the animals				
Fever	45%	44%	48%	40%
Sweats	15%	16%	16%	12%
Malaise	31%	20%	20%	52%
Headache	57%	60%	48%	64%
Pain in muscles, joints and/or back	57%	56%	52%	64%

All farms had at least five risk factors reported, with a higher average in Mara Rianta (7.6), than Lemek (7.0) and Endoinyo Narasha (6.8). Seropositive farms generally did have a higher risk score (average 7.4) than negative farms (average 7.1) but the difference was not significant (Table 3).

## Discussion

More than a fifth of the farms and a tenth of the cattle had evidence of past *Brucella* infection in this study, with higher odds of animals being positive closer to the national reserve. To our knowledge, no similar study has been carried out on *Brucella* in this context. In addition, both livestock and humans had experienced symptoms that are consistent with the pathogen. Although this was not assessed in this study, it is possible that some clinical signs were caused by brucellosis. Indeed, human *Brucella* infections and disease have been previously reported in Kenya [9, 10].

There may be different explanations to the higher seroprevalence in Mara Rianta observed here. While all sites practices natural breeding, which has been associated with higher risk of brucellosis [11], all three positives males were in Mara Rianta, which may indicate a higher infection rate in the males in that population, further increasing the risks for the females. However, due to the small number of males sampled in this study, further research is needed to conclude on this. It is likely that a larger herd size entails a greater risk for *Brucella* infection. Previous studies suggest small, confined herds have lower risks [3], which is supported by the results from this study where Endoinyo Narasha was the village with the lowest prevalence, the smallest herds, and the only village where cattle farms were fenced.

There is a possibility that cattle in the infected farms have had abortions earlier since most cattle only abort once after infection [5], and we found a tendency among positive farms to report a higher degree of reproductive problems. There are many reasons why cattle may abort, and many of these are infectious [12]. Further studies are needed to investigate the relative importance of *Brucella* spp. as a cause of abortion in cattle of the examined area. Similarly the symptoms reported by farmers may be caused by many different diseases, and further studies would be required to provide more insight knowledge on the contribution of different pathogens to the total disease burden.

Wildlife can be a source of infectious diseases and thereby a risk to the health of the livestock as has been shown for some diseases [13]. A significant difference was found in prevalence of brucellosis between the villages, with cattle from Mara Rianta having the highest proportion of positive animals. These animals were grazing inside the national reserve Maasai Mara, and interacting with wildlife to a higher degree, than cattle that mostly graze behind fences within the own village, as in Endoinyo Narasha. *Brucella* exposure has been reported in both African buffaloes (*Syncerus caffer*), blue wildebeest (*Connochaetes taurinus*), oryx (*Oryx beisa*) and eland (*Taurotragus oryx*); common in and around Maasai Mara [14, 15].

Brucellosis remains an important problem in Kenya since there is currently no possibility of controlling or eradicating the disease the same way as in high-income countries, and vaccination is not common. None of the participating farmers reported that they had vaccinated their animals against the disease. In Kenya, a suitable method of controlling disease has to be introduced, with components of disease surveillance, mapping of transmission routes, vaccination campaigns, isolation of exposed animals and thorough examination before introducing new individuals to a healthy animal herd ought to be fundamental since all mentioned factors are important cornerstones in successful disease control. Since there are no vaccines available for humans, prevention of brucellosis in humans relies fully in controlling the animal reservoir.

## Limitations

This study had several limitations, including the limited sample size of 75 herds, and few males sampled. Although selection of animals at herd level was intended to be unbiased and random, large bulls and aggressive individuals were excluded; however, the authors are not expecting this to influence the results significantly. There were many confounders in the study, and herd size was much depending on the area of sampling, meaning that it is difficult to draw definite conclusions on these factors. To fully evaluate the impact on human health, it would be necessary to collect data on incidence and morbidity.

## Additional files

**Additional file 1.** Questionnaire. This file is containing the questions for the interviews with farmers.

**Additional file 2.** Additional table with all data. This file is containing all data collected and analyzed.

## Abbreviations

CI: confidence interval
ELISA: enzyme-linked immunosorbent assay
IACUC: Institutional Animal Care and Use Committee
ILRI: International Livestock Research Institute
OR: odds ratio
