# Supplementary material for: Brucella seroprevalence in cattle near a wildlife reserve in Kenya
Source: BMC Res Notes. 2017 Nov 25;10:615. doi: 10.1186/s13104-017-2941-x (PMC5702115; doi:10.1186/s13104-017-2941-x)
Supplement: Supplementary file 1 — Additional file 1. Questionnaire. This file is containing the questions for the interviews with farmers. [file 13104_2017_2941_MOESM1_ESM.docx]

**Interview questions for *Brucella* seroprevalence study**

1. Do you have communal grazing areas/ grazing reserves within the village?
   1. Yes b. No
2. Do farmers within the village share these grazing areas?
   1. Yes b. No
3. Do farmers from other villages share the communal grazing areas?
   1. Yes b. No

If yes, when? ______________________________________________

1. Do your cattle graze in the national park?
   1. Yes b. No
2. Does your livestock mix with other herds in the grazing areas?
   1. Yes b. No
3. How many herds do they mix with while grazing?
   1. Daily: _______ b. Weekly: _______ c. Monthly: _______
4. Do you sight wildlife near your livestock at grazing or when on transhumance?
   1. Yes b. No
5. Which types of wildlife do you see near your livestock?
   1. Ungulates b. Predators c. Monkeys d. Other.
6. How do you experience the contact with wildlife?
   1. Positive b. Negative c. Indifferent.
7. Which species of livestock do you see?
   1. Goats b. Sheep c. Pigs d. Poultry e. Other
8. Do you have a water source for your herd within the farm?
   1. Yes b. No
9. Are these water sources shared with other herds within the village?
   1. Yes b. No
10. Do neighboring villages share these watering points?
    1. Yes b. No
11. How many other herds use this watering point for their livestock?
    1. 1-5 b. 5-10 c. 10-15 d. 15-20 e. >20

f. None

1. Do your cattle mix with other herds at watering point? How many herds do they mix with when they drink?
   1. Yes b. No

If yes, how many?: _____________________________________________

1. Does your livestock share trek routes with herds?
   1. Yes b. No c. Unsure
2. Do your herd share water points on the trek with other herds?
   1. Yes b. No c. Unsure
3. Did you buy any livestock in the last one year? How many, species and which market?
   1. Yes b. No

If yes, please specify: ____________________________________________

1. Type of husbandry eg breeding bull, AI, breeding bull own, common use, breeding bull from another farm etc

Please specify: __________________________________________________

1. Have you noticed any of the following signs of illness in your cattle? (Choose as many as needed):
   1. Fatigue
   2. Loss of pregnancy/abortion/stillbirth
   3. Decrease in milk production
   4. Mastitis/udder swelling and/or pain
   5. Unwillingness to walk/stand
   6. Fever
   7. Blisters in mouth, teats or hooves?
2. Have any of the people handling the animals experienced:
   1. Fever
   2. Sweat
   3. Malaise
   4. Headache
   5. Pain in muscles, joints and/or back

(Choose as many as needed)
